# Supplementary material for: Standardized Diagnostic Assays for Omsk Hemorrhagic Fever Virus
Source: Pathogens. 2025 Oct 27;14(11):1093. doi: 10.3390/pathogens14111093 (PMC12655418; doi:10.3390/pathogens14111093)
Supplement: Supplementary file 1 [file pathogens-14-01093-s001.zip › pathogens-3936452-supplementary.pdf]

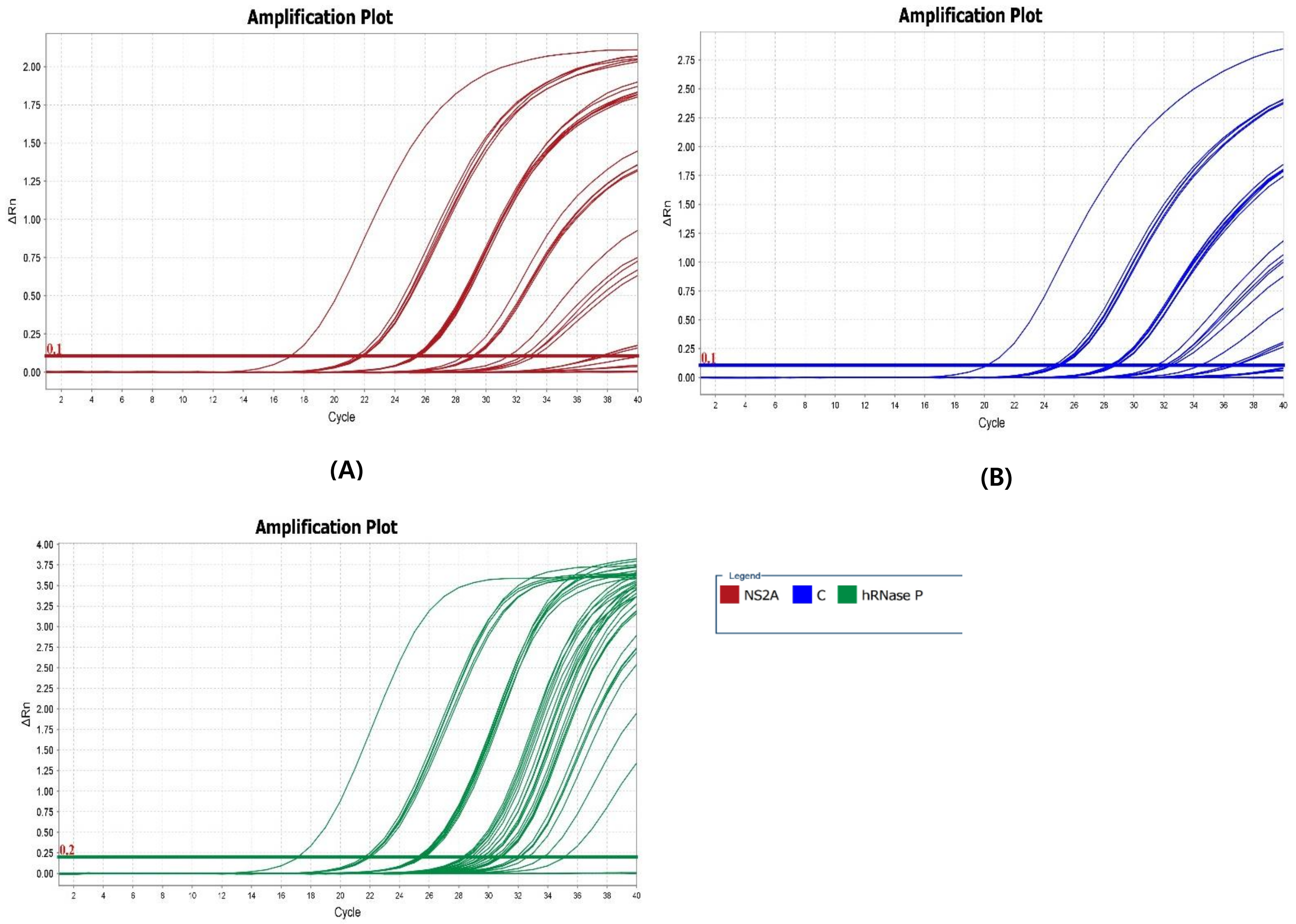

Supplementary Figure S1. Representative amplification plots for the OHFV real-time RT-PCR assay.

(A) NS2A (FAM, red), (B) C (VIC, blue), and (C) hRNase P (Cy5, green) amplification plots. Ten-fold dilutions from 10^5 to 10^2 copies/µL with NTC are shown. The threshold (0.1 dRn, horizontal line) was placed in the exponential phase, and the baseline was set automatically by the software (QuantStudio™ Dx v1.3). Data at 10^1 copies/µL were excluded from linearity assessment due to partial/non-detection.

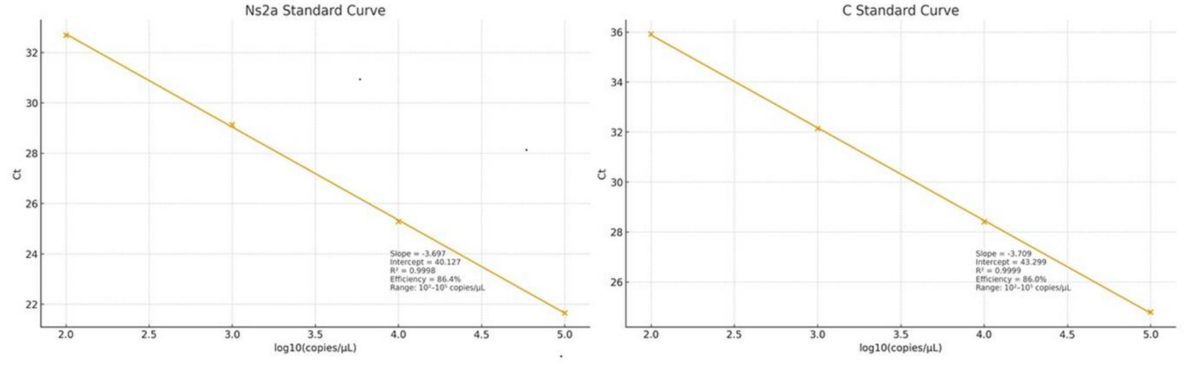

Supplementary Figure S2. Standard curves for the OHFV real-time RT-PCR assay.

(A) NS2A and (B) C standard curves generated from the 10-fold dilution series (10^2–10^5 copies/µL). Linear regression of Ct versus

log_{10}(input copies) gave slopes of −3.697 (NS2A) and −3.709 (C) with R^2 = 0.9998 and 0.9999, corresponding to amplification efficiencies of 86.4% and 86.0%, respectively. Data at 10^1 copies/μL were excluded due to partial/non-detection; therefore, the linear dynamic range was 10^2–10^5 copies/μL. Points represent the mean Ct of four replicates (error bars omitted for clarity).

Supplementary Table S1. Criteria for interpreting real-time PCR results.

Case		Target gene Ct result	Positive control	Negative control	Internal control (IC)*	Interpretation	
1	*NS2A*	< 32.99	+	-	+	Positive	
	C	< 35.38	+	-			
2	*NS2A*	< 32.99	+	-	-	Retest	
	C	< 35.38	+	-			
3	*NS2A*	> 35.04	+	-	+	Negative	
	C	> 37.04	+	-			
4	*NS2A*	> 35.04	+	-	-	Retest (recollection)**	
	C	> 37.04	+	-			
5	*NS2A*	32.99 ≤ Ct value ≤ 35.04	+	-	+	Inconclusive	Retest (recollection)
	C	35.38≤ Ct value ≤ 37.04	+	-			
6	*NS2A*	32.99 ≤ Ct value ≤ 35.04	+	-	-		
	C	35.38≤ Ct value ≤ 37.04	+	-			
7	*NS2A*	< 32.99	+	-	+	Retest	
	C	> 37.04	+	-			
8	*NS2A*	< 32.99	+	-	-		
	C	> 37.04	+	-			
9	*NS2A*	> 35.04	+	-	+		
	C	< 35.38	+	-			
10	*NS2A*	> 35.04	+	-	-		
	C	< 35.38	+	-			
11	*NS2A*	±	+	+	±	Negative control failure	Retest (re-extraction)***
	C	±	+	+			
12	*NS2A*	±	-	-	±	Positive control failure	
	C	±	-	-			
13	*NS2A*	±	-	+	±	Positive/Negative control failure	
	C	±	-	+			

* Internal control: In most cases, IC is positive when nucleic acids are extracted from clinical specimens. However, depending on the condition and type of specimen, IC may be negative. For example, when target nucleic acids are strongly positive, IC amplification may be inhibited or undetected; in such cases, specimens are diluted and retested by real-time PCR or recollected and re-examined.
** Retest required after recollection of specimen.
*** Replace operator, reagents, and positive/negative controls, and repeat RNA extraction.

Supplementary Table S2. Validation Specimen List and Quantification Values

No.	**Sample ID**	**Quantification (copies/μL)**
1	Panel 1-1	1×10^5 copies/μL
2	Panel 1-2	1×10^4 copies/μL
3	Panel 1-3	1×10^3 copies/μL
4	Panel 1-4	1×10^2 copies/μL
5	Panel 1-5	1×10^1 copies/μL
6	Panel 1-6	1×10^0 copies/μL
7	Panel 1-7	1×10^{-1} copies/μL
9	Panel 2-1	100 copies/μL
10	Panel 2-2	80 copies/μL
11	Panel 2-3	60 copies/μL
12	Panel 2-4	40 copies/μL
13	Panel 2-5	20 copies/μL
14	Panel 2-6	0 copies/μL
16	Panel 3-1	50.24 copies/μL
17	Panel 3-2	46.44 copies/μL
18	Inconclusive 1-1	74.50 copies/μL
19	Inconclusive 1-2	70.41 copies/μL
20	Inconclusive 2-1	33.88 copies/μL
21	Inconclusive 2-2	30.63 copies/μL

Note: Synthetic RNAs corresponding to the NS2A and C genes of Omsk hemorrhagic fever virus were prepared at the indicated concentrations, and an internal control was spiked into all contrived specimens at 1×10^3 copies/μL.

Supplementary Table S3. Preliminary Results for Establishing Cutoff Thresholds

**Specimen ID**	**Target (Fluorophore)**	**Threshold Cycle (Ct)**				**Mean Ct.**	**Detection rate (%)**
		**1st**	**2nd**	**3rd**	**4th**		
Panel 1-1	NS2A (FAM)	21.42	21.67	21.69	21.82	21.65	100 (4/4)
	C(VIC)	24.52	24.77	24.90	25.00	24.80	100 (4/4)

	IC(Cy5)	27.46	24.43	31.19	24.59	26.91	100 (4/4)
Panel 1-2	NS2A (FAM)	25.19	25.25	25.33	25.34	25.28	100 (4/4)
	C(VIC)	28.31	28.42	28.43	28.47	28.41	100 (4/4)
	IC(Cy5)	32.74	29.34	29.63	24.37	29.02	100 (4/4)
Panel 1-3	NS2A (FAM)	29.02	29.12	29.14	29.23	29.13	100 (4/4)
	C(VIC)	32.41	31.97	31.97	32.21	32.14	100 (4/4)
	IC(Cy5)	24.22	21.00	27.36	27.52	25.02	100 (4/4)
Panel 1-4	NS2A (FAM)	32.25	32.71	33.09	32.71	32.69	100 (4/4)
	C(VIC)	34.31	36.16	36.56	36.65	35.92	100 (4/4)
	IC(Cy5)	29.92	28.27	28.99	28.97	29.04	100 (4/4)
Panel 1-5	NS2A (FAM)	37.31	-*	37.68	-	37.50	50 (2/4)
	C(VIC)	-	-	-	-	-	0 (0/4)
	IC(Cy5)	20.57	30.78	24.27	28.52	26.04	100 (4/4)
Panel 1-6	NS2A (FAM)	-	-	-	-	-	0 (0/4)
	C(VIC)	-	-	-	-	-	0 (0/4)
	IC(Cy5)	27.25	27.79	29.74	24.27	27.26	100 (4/4)
Panel 1-7	NS2A (FAM)	–	–	–	–	–	0 (0/4)
	C(VIC)	–	–	–	–	–	0 (0/4)
	IC(Cy5)	31.18	34.07	29.82	24.56	29.91	100 (4/4)
Positive control	NS2A (FAM)	17.04					
	C(VIC)	20.10					
	IC(Cy5)	27.33					
Negative control	NS2A (FAM)	-					
	C(VIC)	-					
	IC(Cy5)	–					

* : Not Detected

Supplementary Table S4. Probit analysis.

Specimen ID	Target (Fluorophore)	Concentration (copies/μL)	Detected/n	Detection rate (%)	Mean Ct	
					OHFV	IC
Panel 2-1	*NS2A* (FAM)	100	10/10	100	31.05	29.72
	C (VIC)	100	10/10	100	34.50	
Panel 2-2	*NS2A* (FAM)	80	10/10	100	31.79	31.06
	C (VIC)	80	10/10	100	35.29	
Panel 2-3	*NS2A* (FAM)	60	7/10	70	32.76	30.47
	C (VIC)	60	8/10	80	36.25	
Panel 2-4	*NS2A* (FAM)	40	2/10	20	33.64	29.55
	C (VIC)	40	3/10	30	36.71	
Panel 2-5	*NS2A* (FAM)	20	0/10	0.0	-*	30.41
	C (VIC)	20	0/10	0.0	-	
Panel 2-6	*NS2A* (FAM)	0	0/10	0.0	-	27.06
	C (VIC)	0	0/10	0.0	-	
Positive control	*NS2A* (FAM)	17.12	IC	25.35		
	C (VIC)	18.07				
Negative control	*NS2A* (FAM)	-	IC	-		
	C (VIC)	-				

Specimen ID	Target	Concentration (copies/μL)	Detected (n)	Mean Ct. OHFV	IC	Inconclusive Zone
Inconclusive 1-2	*NS2A* (FAM)	74.50	39/40	32.99	30.81	Upper
Inconclusive 1-1	*C* (VIC)	70.41	38/40	35.38		
Inconclusive 2-1	*NS2A* (FAM)	33.88	2/40	35.04	30.78	Lower
Inconclusive 2-2	*C* (VIC)	30.63	2/40	37.04		

NS2A (FAM) Probit analysis result

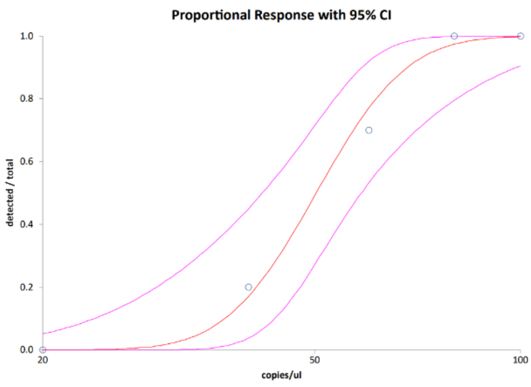

C (VIC) Probit analysis result

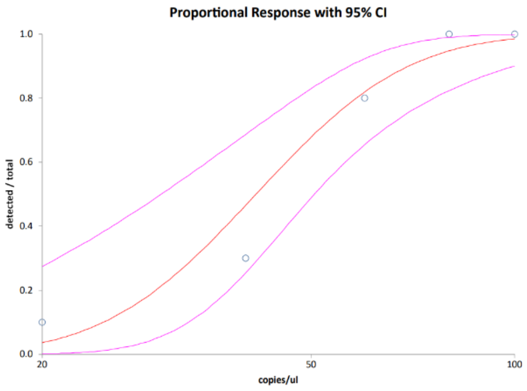

**Probit analysis - probit sigmoid curve**<br><br>constant = -16.355327<br>slope = 9.614632<br>Median * Dose = 50.244334<br>Confidence interval (No Heterogeneity) = 41.372076 to 58.164278<br>* Dose for centile 95 = 74.50154<br>Confidence interval (No Heterogeneity) = 63.14421 to 114.542844<br>Chi^2 (heterogeneity of deviations from model) = 0.632175 (3 df) P = 0.889<br>t for slope = 3.677168 (3 df_slope) P = 0.0348<br>* Dose for centile 5 = 33.885113<br>Confidence interval (No Heterogeneity) = 19.427021 to 41.211613<br>Chi^2 (heterogeneity of deviations from model) = 0.632175 (3 df) P = 0.889<br>t for slope = 3.677168 (3 df_slope) P = 0.0348	**Probit analysis - probit sigmoid curve**<br><br>constant = -15.174064<br>slope = 9.1029<br>Median * Dose = 46.446011<br>Confidence interval (No Heterogeneity) = 36.882989 to 54.214642<br>* Dose for centile 95 = 70.411563<br>Confidence interval (No Heterogeneity) = 59.111089 to 116.004801<br>Chi^2 (heterogeneity of deviations from model) = 0.351783 (3 df) P = 0.95<br>t for slope = 3.439286 (3 df_slope) P = 0.0413<br>* Dose for centile 5 = 30.637466<br>Confidence interval (No Heterogeneity) = 15.318143 to 38.065748<br>Chi^2 (heterogeneity of deviations from model) = 0.351783 (3 df) P = 0.95<br>t for slope = 3.439286 (3 df_slope) P = 0.0413
C_{95}: 74.50copies/μL (95% CI 63.14~ 114.54)<br>C_{50}: 50.24 copies/μL (95% CI 41.37 ~ 58.16)<br>C_{5}: 33.88 copies/μL (95% CI 19.42 ~ 41.21)	C_{95}: 70.41 copies/μL (95% CI 59.11 ~ 116.00)<br>C_{50}: 46.44 copies/μL (95% CI 36.88 ~ 54.21)<br>C_{5}: 30.63 copies/μL (95% CI 15.31~ 38.06)

* : Not Detected

Supplementary Table S5. Results for establishing inconclusive zones.

Specimen ID	Target	Concentration (copies/μL)	Detected (n)	Mean Ct. OHFV	Mean Ct. IC	Inconclusive Zone
Inconclusive 1-2	*NS2A* (FAM)	74.50	39/40	32.99	30.81	Upper
Inconclusive 1-1	*C* (VIC)	70.41	38/40	35.38		
Inconclusive 2-1	*NS2A* (FAM)	33.88	2/40	35.04	30.78	Lower
Inconclusive 2-2	*C* (VIC)	30.63	2/40	37.04		

Supplementary Table S6. Composition of contrived positive and negative specimens

No.	Material name	Specimen type	Detection method	Quantification value (copies/μL)	Source
1	AMPLIRUN® WEST NILE VIRUS RNA CONTROL 1x10² copies/μL	Synthetic RNA	Real-time PCR	-*	AMPLIRUN
2	AMPLIRUN® WESTERN EQUINE ENCEPHALITIS RNA CONTROL 1x10² copies/μL	Synthetic RNA	Real-time PCR	-	AMPLIRUN
3	AMPLIRUN® YELLOW FEVER VIRUS RNA CONTROL 1x10² copies/μL	Synthetic RNA	Real-time PCR	-	AMPLIRUN
4	AMPLIRUN® ZIKA VIRUS (ASIAN LINEAGE) RNA CONTROL 1x10² copies/μL	Synthetic RNA	Real-time PCR	-	AMPLIRUN
5	AMPLIRUN® ZIKA VIRUS RNA CONTROL 1x10² copies/μL	Synthetic RNA	Real-time PCR	-	AMPLIRUN
6	AMPLIRUN® ST LOUIS ENCEPHALITIS VIRUS RNA CONTROL 1x10² copies/μL	Synthetic RNA	Real-time PCR	-	AMPLIRUN
7	AMPLIRUN® TICK BORNE ENCEPHALITIS VIRUS RNA CONTROL 1x10² copies/μL	Synthetic RNA	Real-time PCR	-	AMPLIRUN

No.	Material name	Specimen type	Detection method	Quantification value (copies/μL)	Source
8	AMPLIRUN® VENEZUELAN EQUINE ENCEPHALITIS RNA CONTROL 1x10² copies/μL	Synthetic RNA	Real-time PCR	-	AMPLIRUN
9	Omsk hemorrhagic fever virus positive material 3× LOD-1	Synthetic RNA	Real-time PCR	N(223.50)<br>C(211.23)	ATTOPLEX
10	Omsk hemorrhagic fever virus positive material 3 X LOD-2	Synthetic RNA	Real-time PCR	N(223.50)<br>C(211.23)	ATTOPLEX
11	Omsk hemorrhagic fever virus positive material 3 X LOD-3	Synthetic RNA	Real-time PCR	N(223.50)<br>C(211.23)	ATTOPLEX
12	Omsk hemorrhagic fever virus positive material 3 X LOD-4	Synthetic RNA	Real-time PCR	N(223.50)<br>C(211.23)	ATTOPLEX
13	Omsk hemorrhagic fever virus positive material 3 X LOD-5	Synthetic RNA	Real-time PCR	N(223.50)<br>C(211.23)	ATTOPLEX
14	Omsk hemorrhagic fever virus positive material 1.5 X LOD-1	Synthetic RNA	Real-time PCR	N(111.75)<br>C(105.61)	ATTOPLEX
15	Omsk hemorrhagic fever virus positive material 1.5 X LOD-2	Synthetic RNA	Real-time PCR	N(111.75)<br>C(105.61)	ATTOPLEX
16	Omsk hemorrhagic fever virus positive material 1.5 X LOD-3	Synthetic RNA	Real-time PCR	N(111.75)<br>C(105.61)	ATTOPLEX
17	Omsk hemorrhagic fever virus positive material 1.5 X LOD-4	Synthetic RNA	Real-time PCR	N(111.75)<br>C(105.61)	ATTOPLEX
18	Omsk hemorrhagic fever virus positive material 1.5 X LOD-5	Synthetic RNA	Real-time PCR	N(111.75)<br>C(105.61)	ATTOPLEX
19	AMPLIRUN® CHIKUNGUNYA VIRUS RNA CONTROL 1x10² copies/μL	Synthetic RNA	Real-time PCR	-	AMPLIRUN
20	AMPLIRUN® DENGUE 1 VIRUS RNA CONTROL 1x10² copies/μL	Synthetic RNA	Real-time PCR	-	AMPLIRUN
21	AMPLIRUN® DENGUE 2 VIRUS RNA CONTROL 1x10² copies/μL	Synthetic RNA	Real-time PCR	-	AMPLIRUN
22	AMPLIRUN® DENGUE 3 VIRUS RNA CONTROL 1x10² copies/μL	Synthetic RNA	Real-time PCR	-	AMPLIRUN
23	Omsk hemorrhagic fever virus positive material 1.5 X LOD-6	Synthetic RNA	Real-time PCR	N(111.75)<br>C(105.61)	ATTOPLEX
24	Omsk hemorrhagic fever virus positive material 1.5 X LOD-7	Synthetic RNA	Real-time PCR	N(111.75)<br>C(105.61)	ATTOPLEX
25	Omsk hemorrhagic fever virus positive material 1.5 X LOD-8	Synthetic RNA	Real-time PCR	N(111.75)<br>C(105.61)	ATTOPLEX
26	Omsk hemorrhagic fever virus positive material 1.5 X LOD-9	Synthetic	Real-time	N(111.75)	ATTOPLEX

No.	Material name	Specimen type	Detection method	Quantification value (copies/μL)	Source
		RNA	PCR	C(105.61)	
27	Omsk hemorrhagic fever virus positive material 1.5 X LOD-10	Synthetic RNA	Real-time PCR	N(111.75)<br>C(105.61)	ATTOPLEX
28	AMPLIRUN® DENGUE 4 VIRUS RNA CONTROL 1x10² copies/μL	Synthetic RNA	Real-time PCR	-	AMPLIRUN
29	AMPLIRUN® EASTERN EQUINE ENCEPHALITIS RNA CONTROL 1x10² copies/μL	Synthetic RNA	Real-time PCR	-	AMPLIRUN
30	EBOV RNA NP-VP40-L, WHO Reference Reagent 1x10² pg/μL	Synthetic RNA	Real-time PCR	-	NIBSC
31	EBOV RNA NP-VP35-GP, WHO Reference Reagent 1x10² pg/μL	Synthetic RNA	Real-time PCR	-	NIBSC
32	AMPLIRUN® ST LOUIS ENCEPHALITIS VIRUS RNA CONTROL 2x10² copies/μL	Synthetic RNA	Real-time PCR	-	AMPLIRUN
33	Omsk hemorrhagic fever virus positive material 3XLOD-14	Synthetic RNA	Real-time PCR	N(223.50)<br>C(211.23)	ATTOPLEX
34	Omsk hemorrhagic fever virus positive material 3XLOD-15	Synthetic RNA	Real-time PCR	N(223.50)<br>C(211.23)	ATTOPLEX
35	Omsk hemorrhagic fever virus positive material 3XLOD-16	Synthetic RNA	Real-time PCR	N(223.50)<br>C(211.23)	ATTOPLEX
36	Omsk hemorrhagic fever virus positive material 3XLOD-17	Synthetic RNA	Real-time PCR	N(223.50)<br>C(211.23)	ATTOPLEX
37	Omsk hemorrhagic fever virus positive material 3XLOD-18	Synthetic RNA	Real-time PCR	N(223.50)<br>C(211.23)	ATTOPLEX
38	*Yersinia enterocolitica* NCCP10246 (DNA) 5 ng/rxn	Genomic DNA	Real-time PCR	-	KDCA
39	*Burkholderia cepacia* NCCP11153 (DNA) 5 ng/rxn	Genomic DNA	Real-time PCR	-	KDCA
40	*Clostridium difficile* NCCP10868 (DNA) 5 ng/rxn	Genomic DNA	Real-time PCR	-	KDCA
41	AMPLIRUN® WESTERN EQUINE ENCEPHALITIS RNA CONTROL 2x10² copies/μL	Synthetic RNA	Real-time PCR	-	AMPLIRUN
42	AMPLIRUN® YELLOW FEVER VIRUS RNA CONTROL 2x10² copies/μL	Synthetic RNA	Real-time PCR	-	AMPLIRUN
43	AMPLIRUN® ZIKA VIRUS (ASIAN LINEAGE) RNA CONTROL 2x10² copies/μL	Synthetic RNA	Real-time PCR	-	AMPLIRUN
44	AMPLIRUN® ZIKA VIRUS RNA CONTROL 2x10² copies/μL	Synthetic RNA	Real-time PCR		AMPLIRUN

No.	Material name	Specimen type	Detection method	Quantification value (copies/μL)	Source
45	AMPLIRUN® CHIKUNGUNYA VIRUS RNA CONTROL $2x10^2$ copies/μL	Synthetic RNA	Real-time PCR		AMPLIRUN
46	AMPLIRUN® DENGUE 1 VIRUS RNA CONTROL $2x10^2$ copies/μL	Synthetic RNA	Real-time PCR		AMPLIRUN
47	AMPLIRUN® DENGUE 2 VIRUS RNA CONTROL $2x10^2$ copies/μL	Synthetic RNA	Real-time PCR		AMPLIRUN
48	AMPLIRUN® DENGUE 3 VIRUS RNA CONTROL $2x10^2$ copies/μL	Synthetic RNA	Real-time PCR		AMPLIRUN
49	Omsk hemorrhagic fever virus positive material 3 X LOD-6	Synthetic RNA	Real-time PCR	N(223.50)<br>C(211.23)	ATTOPLEX
50	Omsk hemorrhagic fever virus positive material 3 X LOD-7	Synthetic RNA	Real-time PCR	N(223.50)<br>C(211.23)	ATTOPLEX
51	Omsk hemorrhagic fever virus positive material 3 X LOD-8	Synthetic RNA	Real-time PCR	N(223.50)<br>C(211.23)	ATTOPLEX
52	Omsk hemorrhagic fever virus positive material 3 X LOD-9	Synthetic RNA	Real-time PCR	N(223.50)<br>C(211.23)	ATTOPLEX
53	Omsk hemorrhagic fever virus positive material 3 X LOD-10	Synthetic RNA	Real-time PCR	N(223.50)<br>C(211.23)	ATTOPLEX
54	Omsk hemorrhagic fever virus positive material 3 X LOD-11	Synthetic RNA	Real-time PCR	N(223.50)<br>C(211.23)	ATTOPLEX
55	Omsk hemorrhagic fever virus positive material 3 X LOD-12	Synthetic RNA	Real-time PCR	N(223.50)<br>C(211.23)	ATTOPLEX
56	Omsk hemorrhagic fever virus positive material 3 X LOD-13	Synthetic RNA	Real-time PCR	N(223.50)<br>C(211.23)	ATTOPLEX
57	AMPLIRUN® DENGUE 4 VIRUS RNA CONTROL $2x10^2$ copies/μL	Synthetic RNA	Real-time PCR	-	AMPLIRUN
58	AMPLIRUN® EASTERN EQUINE ENCEPHALITIS RNA CONTROL $2x10^2$ copies/μL	Synthetic RNA	Real-time PCR	-	AMPLIRUN
59	*Escherichia coli* NCCP11157 (DNA) 5 ng/rxn	Genomic DNA	Real-time PCR	-	KDCA
60	*Campylobacter jejuni* NCCP10402 (DNA) 5 ng/rxn	Genomic DNA	Real-time PCR	-	KDCA
61	*Brucella abortus* NCCP10040 (DNA) 5 ng/rxn	Genomic DNA	Real-time PCR	-	KDCA
62	Omsk hemorrhagic fever virus positive material 1.5 X LOD-11	Synthetic RNA	Real-time PCR	N(111.75)<br>C(105.61)	ATTOPLEX
63	Omsk hemorrhagic fever virus positive material 1.5 X LOD-12	Synthetic RNA	Real-time PCR	N(111.75)<br>C(105.61)	ATTOPLEX

No.	Material name	Specimen type	Detection method	Quantification value (copies/μL)	Source
64	Omsk hemorrhagic fever virus positive material 1.5 X LOD-13	Synthetic RNA	Real-time PCR	N(111.75)<br>C(105.61)	ATTOPLEX
65	Omsk hemorrhagic fever virus positive material 1.5 X LOD-14	Synthetic RNA	Real-time PCR	N(111.75)<br>C(105.61)	ATTOPLEX
66	Omsk hemorrhagic fever virus positive material 1.5 X LOD-15	Synthetic RNA	Real-time PCR	N(111.75)<br>C(105.61)	ATTOPLEX
67	Omsk hemorrhagic fever virus positive material 3XLOD-19	Synthetic RNA	Real-time PCR	N(223.50)<br>C(211.23)	ATTOPLEX
68	Omsk hemorrhagic fever virus positive material 3XLOD-20	Synthetic RNA	Real-time PCR	N(223.50)<br>C(211.23)	ATTOPLEX
69	EBOV RNA NP-VP40-L, WHO Reference Reagent $2x10^2$ pg/μL	Synthetic RNA	Real-time PCR	-	NIBSC
70	EBOV RNA NP-VP35-GP, WHO Reference Reagent $2x10^2$ pg/μL	Synthetic RNA	Real-time PCR	-	NIBSC
71	AMPLIRUN® TICK BORNE ENCEPHALITIS VIRUS RNA CONTROL $1x10^3$ copies/μL	Synthetic RNA	Real-time PCR	-	AMPLIRUN
72	AMPLIRUN® YELLOW FEVER VIRUS RNA CONTROL $1x10^3$ copies/μL	Synthetic RNA	Real-time PCR	-	AMPLIRUN
73	AMPLIRUN® EASTERN EQUINE ENCEPHALITIS RNA CONTROL $1x10^3$ copies/μL	Synthetic RNA	Real-time PCR	-	AMPLIRUN
74	EBOV RNA NP-VP40-L, WHO Reference Reagent $1x10^3$ copies/μL	Synthetic RNA	Real-time PCR	-	NIBSC
75	*Bacillus subtilis* NCCP10857(DNA)	Genomic DNA	Real-time PCR	-	KDCA
76	*Bacillus cereus* NCCP10070(DNA) 5 ng/rxn	Genomic DNA	Real-time PCR	-	KDCA
77	Omsk hemorrhagic fever virus positive material 1.5XLOD-18	Synthetic RNA	Real-time PCR	N(111.75)<br>C(105.61)	ATTOPLEX
78	Omsk hemorrhagic fever virus positive material 1.5XLOD-19	Synthetic RNA	Real-time PCR	N(111.75)<br>C(105.61)	ATTOPLEX
79	Omsk hemorrhagic fever virus positive material 1.5XLOD-20	Synthetic RNA	Real-time PCR	N(111.75)<br>C(105.61)	ATTOPLEX
80	AMPLIRUN® VENEZUELAN EQUINE ENCEPHALITIS RNA CONTROL $1x10^3$ copies/μL	Synthetic RNA	Real-time PCR	-	AMPLIRUN
81	AMPLIRUN® WEST NILE VIRUS RNA CONTROL $1x10^3$ copies/μL	Synthetic RNA	Real-time PCR	-	AMPLIRUN
82	AMPLIRUN® TICK BORNE ENCEPHALITIS VIRUS RNA CONTROL $2x10^2$ copies/μL	Synthetic RNA	Real-time PCR	-	AMPLIRUN

No.	Material name	Specimen type	Detection method	Quantification value (copies/μL)	Source
83	AMPLIRUN® VENEZUELAN EQUINE ENCEPHALITIS RNA CONTROL 2x10² copies/μL	Synthetic RNA	Real-time PCR	-	AMPLIRUN
84	AMPLIRUN® WEST NILE VIRUS RNA CONTROL 2x10² copies/μL	Synthetic RNA	Real-time PCR	-	AMPLIRUN
85	*Vibrio cholerae* NAG NCCP11179 (DNA) 5 ng/rxn	Synthetic RNA	Real-time PCR	-	KDCA
86	AMPLIRUN® WESTERN EQUINE ENCEPHALITIS RNA CONTROL 1x10³ copies/μL	Synthetic RNA	Real-time PCR	-	AMPLIRUN
87	Omsk hemorrhagic fever virus positive material 1.5XLOD-16	Synthetic RNA	Real-time PCR	N(111.75)<br>C(105.61)	ATTOPLEX
88	Omsk hemorrhagic fever virus positive material 1.5XLOD-17	Synthetic RNA	Real-time PCR	N(111.75)<br>C(105.61)	ATTOPLEX
89	Omsk hemorrhagic fever virus positive material 3XLOD-21	Synthetic RNA	Real-time PCR	N(223.50)<br>C(211.23)	ATTOPLEX
90	Omsk hemorrhagic fever virus positive material 3 XLOD-22	Synthetic RNA	Real-time PCR	N(223.50)<br>C(211.23)	ATTOPLEX
91	Omsk hemorrhagic fever virus positive material 3 XLOD-23	Synthetic RNA	Real-time PCR	N(223.50)<br>C(211.23)	ATTOPLEX
92	Omsk hemorrhagic fever virus positive material 1.5 XLOD-21	Synthetic RNA	Real-time PCR	N(111.75)<br>C(105.61)	ATTOPLEX
93	Omsk hemorrhagic fever virus positive material 1.5 XLOD-22	Synthetic RNA	Real-time PCR	N(111.75)<br>C(105.61)	ATTOPLEX
94	Omsk hemorrhagic fever virus positive material 1.5 XLOD-23	Synthetic RNA	Real-time PCR	N(111.75)<br>C(105.61)	ATTOPLEX
95	Omsk hemorrhagic fever virus positive material 1.5 XLOD-24	Synthetic RNA	Real-time PCR	N(111.75)<br>C(105.61)	ATTOPLEX
96	Omsk hemorrhagic fever virus positive material 1.5 XLOD-25	Synthetic RNA	Real-time PCR	N(111.75)<br>C(105.61)	ATTOPLEX
97	Omsk hemorrhagic fever virus positive material 3XLOD-24	Synthetic RNA	Real-time PCR	N(223.50)<br>C(211.23)	ATTOPLEX
98	Omsk hemorrhagic fever virus positive material 3XLOD-25	Synthetic RNA	Real-time PCR	N(223.50)<br>C(211.23)	ATTOPLEX
99	2025 Jeonbuk Herpes B virus negative specimen-1	Skin lesion fluid	Real-time PCR	-	KDCA
100	*Salmonella typhi* NCCP14641 (DNA) 5 ng/rxn	Genomic DNA	Real-time PCR	-	KDCA

No.	Material name	Specimen type	Detection method	Quantification value (copies/μL)	Source

* : Not Detected

Note: Omsk hemorrhagic fever virus positive material was prepared by mixing synthetic NS2A and C gene materials with the internal control gene hRNase P.

Composition of positive and negative contrived specimens. Positive specimens (n=50) were prepared at 1.5× and 3× LOD using NS2A (111.75 and 223.50 copies/μL) and C (105.61 and 211.23 copies/μL) gene materials combined with the internal control hRNase P (1×10^3 copies/μL), with 25 specimens generated at each concentration. Negative specimens (n=50) were prepared using viral nucleic acids (AMPLIRUN®, NIBSC) and bacterial nucleic acids (KDCA), all mixed with hRNase P as an internal control.

Supplementary Table S7. Summary of assay validation results.

No.	Material name	Confirmed value			Result interpretation
		NS2A	C	IC	
1	AMPLIRUN® WEST NILE VIRUS RNA CONTROL 1×10^2 copies/μL	-*	-	33.65	Negative
2	AMPLIRUN® WESTERN EQUINE ENCEPHALITIS RNA CONTROL 1×10^2 copies/μL	-	-	33.79	Negative
3	AMPLIRUN® YELLOW FEVER VIRUS RNA CONTROL 1×10^2 copies/μL	-	-	33.66	Negative
4	AMPLIRUN® ZIKA VIRUS (ASIAN LINEAGE) RNA CONTROL 1×10^2 copies/μL	-	-	33.51	Negative
5	AMPLIRUN® ZIKA VIRUS RNA CONTROL 1×10^2 copies/μL	-	-	31.73	Negative
6	AMPLIRUN® ST LOUIS ENCEPHALITIS VIRUS RNA CONTROL 1×10^2 copies/μL	-	-	34.84	Negative
7	AMPLIRUN® TICK BORNE ENCEPHALITIS VIRUS RNA CONTROL 1×10^2 copies/μL	-	-	33.79	Negative
8	AMPLIRUN® VENEZUELAN EQUINE ENCEPHALITIS RNA CONTROL 1×10^2 copies/μL	-	-	32.72	Negative
9	Omsk hemorrhagic fever virus positive material 3 X LOD-1	32.06	33.95	31.96	Positive
10	Omsk hemorrhagic fever virus positive material 3 X LOD-2	33.35	34.64	35.18	Positive
11	Omsk hemorrhagic fever virus positive material 3 X LOD-3	33.31	34.19	31.57	Positive
12	Omsk hemorrhagic fever virus positive material 3 X LOD-4	32.99	34.47	30.51	Positive

No.	Material name	Confirmed value			Result interpretation
		NS2A	C	IC	
13	Omsk hemorrhagic fever virus positive material 3 X LOD-5	32.94	33.96	33.34	Positive
14	Omsk hemorrhagic fever virus positive material 1.5 X LOD-1	33.95	35.47	34.30	Positive
15	Omsk hemorrhagic fever virus positive material 1.5 X LOD-2	34.64	37.85	31.71	Positive
16	Omsk hemorrhagic fever virus positive material 1.5 X LOD-3	34.19	37.62	33.14	Positive
17	Omsk hemorrhagic fever virus positive material 1.5 X LOD-4	34.47	37.58	33.08	Positive
18	Omsk hemorrhagic fever virus positive material 1.5 X LOD-5	33.96	37.34	35.25	Positive
19	AMPLIRUN® CHIKUNGUNYA VIRUS RNA CONTROL 1x10² copies/μL	-	-	34.09	Negative
20	AMPLIRUN® DENGUE 1 VIRUS RNA CONTROL 1x10² copies/μL	-	-	31.51	Negative
21	AMPLIRUN® DENGUE 2 VIRUS RNA CONTROL 1x10² copies/μL	-	-	31.44	Negative
22	AMPLIRUN® DENGUE 3 VIRUS RNA CONTROL 1x10² copies/μL	-	-	32.58	Negative
23	Omsk hemorrhagic fever virus positive material 1.5 X LOD-6	34.96	37.31	34.39	Positive
24	Omsk hemorrhagic fever virus positive material1.5 X LOD-7	34.30	37.30	34.72	Positive
25	Omsk hemorrhagic fever virus positive material 1.5 X LOD-8	34.44	37.00	30.48	Positive
26	Omsk hemorrhagic fever virus positive material 1.5 X LOD-9	34.54	36.95	33.76	Positive
27	Omsk hemorrhagic fever virus positive material 1.5 X LOD-10	34.18	36.71	33.67	Positive
28	AMPLIRUN® DENGUE 4 VIRUS RNA CONTROL 1x10² copies/μL	-	-	34.73	Negative
29	AMPLIRUN® EASTERN EQUINE ENCEPHALITIS RNA CONTROL 1x10² copies/μL	-	-	33.41	Negative
30	EBOV RNA NP-VP40-L, WHO Reference Reagent 1x10² pg/μL	-	-	31.53	Negative
31	EBOV RNA NP-VP35-GP, WHO Reference Reagent 1x10² pg/μL	-	-	31.21	Negative
32	AMPLIRUN® ST LOUIS ENCEPHALITIS VIRUS RNA CONTROL 2x10² copies/μL	-	-	34.12	Negative
33	Omsk hemorrhagic fever virus positive material 3XLOD-14	32.54	34.30	33.20	Positive
34	Omsk hemorrhagic fever virus positive material 3XLOD-15	32.53	34.74	33.34	Positive

No.	Material name	Confirmed value			Result interpretation
		NS2A	C	IC	
35	Omsk hemorrhagic fever virus positive material 3XLOD-16	32.51	34.60	34.39	Positive
36	Omsk hemorrhagic fever virus positive material 3XLOD-17	32.48	34.10	35.47	Positive
37	Omsk hemorrhagic fever virus positive material 3XLOD-18	32.47	34.69	35.72	Positive
38	*Yersinia enterocolitica* NCCP10246 (DNA) 5 ng/rxn	-	-	34.97	Negative
39	*Burkholderia cepacia* NCCP11153 (DNA) 5 ng/rxn	-	-	32.18	Negative
40	*Clostridium difficile* NCCP10868 (DNA) 5 ng/rxn	-	-	33.15	Negative
41	AMPLIRUN® WESTERN EQUINE ENCEPHALITIS RNA CONTROL 2x10^2 copies/μL	-	-	31.46	Negative
42	AMPLIRUN® YELLOW FEVER VIRUS RNA CONTROL 2x10^2 copies/μL	-	-	32.13	Negative
43	AMPLIRUN® ZIKA VIRUS (ASIAN LINEAGE) RNA CONTROL 2x10^2 copies/μL	-	-	35.02	Negative
44	AMPLIRUN® ZIKA VIRUS RNA CONTROL 2x10^2 copies/μL	-	-	33.81	Negative
45	AMPLIRUN® CHIKUNGUNYA VIRUS RNA CONTROL 2x10^2 copies/μL	-	-	32.73	Negative
46	AMPLIRUN® DENGUE 1 VIRUS RNA CONTROL 2x10^2 copies/μL	-	-	32.30	Negative
47	AMPLIRUN® DENGUE 2 VIRUS RNA CONTROL 2x10^2 copies/μL	-	-	35.17	Negative
48	AMPLIRUN® DENGUE 3 VIRUS RNA CONTROL 2x10^2 copies/μL	-	-	31.76	Negative
49	Omsk hemorrhagic fever virus positive material 3 X LOD-6	32.83	34.96	32.99	Positive
50	Omsk hemorrhagic fever virus positive material 3 X LOD-7	32.82	34.30	30.12	Positive
51	Omsk hemorrhagic fever virus positive material 3 X LOD-8	32.79	34.44	30.89	Positive
52	Omsk hemorrhagic fever virus positive material 3 X LOD-9	32.77	34.54	31.56	Positive
53	Omsk hemorrhagic fever virus positive material 3 X LOD-10	32.77	34.18	31.38	Positive
54	Omsk hemorrhagic fever virus positive material 3 X LOD-11	32.71	34.28	31.95	Positive
55	Omsk hemorrhagic fever virus positive material 3 X LOD-12	32.64	34.15	31.26	Positive
56	Omsk hemorrhagic fever virus positive material 3 X LOD-13	32.63	34.61	30.96	Positive

No.	Material name	Confirmed value			Result interpretation
		NS2A	C	IC	
57	AMPLIRUN® DENGUE 4 VIRUS RNA CONTROL $2x10^2$ copies/μL	-	-	30.43	Negative
58	AMPLIRUN® EASTERN EQUINE ENCEPHALITIS RNA CONTROL $2x10^2$ copies/μL	-	-	22.86	Negative
59	*Escherichia coli* NCCP11157 (DNA) 5 ng/rxn	-	-	32.12	Negative
60	*Campylobacter jejuni* NCCP10402 (DNA) 5 ng/rxn	-	-	31.59	Negative
61	*Brucella abortus* NCCP10040 (DNA) 5 ng/rxn	-	-	30.78	Negative
62	Omsk hemorrhagic fever virus positive material 1.5 X LOD-11	34.28	36.71	29.23	Positive
63	Omsk hemorrhagic fever virus positive material 1.5 X LOD-12	34.15	36.58	31.27	Positive
64	Omsk hemorrhagic fever virus positive material 1.5 X LOD-13	34.61	36.53	30.86	Positive
65	Omsk hemorrhagic fever virus positive material 1.5 X LOD-14	34.30	36.37	30.85	Positive
66	Omsk hemorrhagic fever virus positive material 1.5 X LOD-15	34.74	36.36	30.21	Positive
67	Omsk hemorrhagic fever virus positive material3 X LOD-19	32.40	35.03	34.50	Positive
68	Omsk hemorrhagic fever virus positive material 3 X LOD-20	32.38	34.31	29.34	Positive
69	EBOV RNA NP-VP40-L, WHO Reference Reagent $2x10^2$ pg/μL	-	-	30.45	Negative
70	EBOV RNA NP-VP35-GP, WHO Reference Reagent $2x10^2$ pg/μL	-	-	30.91	Negative
71	AMPLIRUN® TICK BORNE ENCEPHALITIS VIRUS RNA CONTROL $1x10^3$ copies/μL	-	-	31.10	Negative
72	AMPLIRUN® YELLOW FEVER VIRUS RNA CONTROL $1x10^3$ copies/μL	-	-	31.50	Negative
73	AMPLIRUN® EASTERN EQUINE ENCEPHALITIS RNA CONTROL $1x10^3$ copies/μL	-	-	30.98	Negative
74	EBOV RNA NP-VP40-L, WHO Reference Reagent $1x10^3$ copies/μL	-	-	28.30	Negative
75	*Bacillus subtilis* NCCP10857(DNA)	-	-	34.27	Negative
76	*Bacillus cereus* NCCP10070(DNA) 5 ng/rxn	-	-	32.19	Negative
77	Omsk hemorrhagic fever virus positive material 1.5 X LOD-18	34.69	36.30	31.77	Positive

No.	Material name	Confirmed value			Result interpretation
		NS2A	C	IC	
78	Omsk hemorrhagic fever virus positive material 1.5 X LOD-19	35.03	36.27	29.04	Positive
79	Omsk hemorrhagic fever virus positive material 1.5 X LOD-20	34.31	36.23	31.11	Positive
80	AMPLIRUN® VENEZUELAN EQUINE ENCEPHALITIS RNA CONTROL $1x10^3$ copies/μL	-	-	30.42	Negative
81	AMPLIRUN® WEST NILE VIRUS RNA CONTROL $1x10^3$ copies/μL	-	-	30.94	Negative
82	AMPLIRUN® TICK BORNE ENCEPHALITIS VIRUS RNA CONTROL $2x10^2$ copies/μL	-	-	31.47	Negative
83	AMPLIRUN® VENEZUELAN EQUINE ENCEPHALITIS RNA CONTROL $2x10^2$ copies/μL	-	-	33.83	Negative
84	AMPLIRUN® WEST NILE VIRUS RNA CONTROL $2x10^2$ copies/μL	-	-	32.19	Negative
85	*Vibrio cholerae* NAG NCCP11179 (DNA) 5 ng/rxn	-	-	31.56	Negative
86	AMPLIRUN® WESTERN EQUINE ENCEPHALITIS RNA CONTROL $1x10^3$ copies/μL	-	-	31.53	Negative
87	Omsk hemorrhagic fever virus positive material 1.5 X LOD-16	34.60	36.34	31.39	Positive
88	Omsk hemorrhagic fever virus positive material 1.5 X LOD-17	34.10	36.31	30.84	Positive
89	Omsk hemorrhagic fever virus positive material 3 X LOD-21	34.69	36.30	30.91	Positive
90	Omsk hemorrhagic fever virus positive material 3 X LOD-22	35.03	36.27	27.48	Positive
91	Omsk hemorrhagic fever virus positive material 3 X LOD-23	34.31	36.23	35.09	Positive
92	Omsk hemorrhagic fever virus positive material 1.5 X LOD-21	34.02	36.02	30.80	Positive
93	Omsk hemorrhagic fever virus positive material 1.5 X LOD-22	34.97	35.96	31.37	Positive
94	Omsk hemorrhagic fever virus positive material 1.5 X LOD-23	35.68	35.81	30.34	Positive
95	Omsk hemorrhagic fever virus positive material 1.5 X LOD-24	34.33	35.62	31.25	Positive
96	Omsk hemorrhagic fever virus positive material 1.5 X LOD-25	34.69	35.52	31.10	Positive
97	Omsk hemorrhagic fever virus positive material 3 X LOD-24	32.19	34.33	30.43	Positive

No.	Material name	Confirmed value			Result interpretation
		NS2A	C	IC	
98	Omsk hemorrhagic fever virus positive material 3 X LOD-25	32.07	34.69	31.70	Positive
99	2025 Jeonbuk Herpes B virus negative specimen-1	-	-	34.82	Negative
100	*Salmonella typhi* NCCP14641 (DNA) 5 ng/rxn	-	-	31.08	Negative
101	Positive control	17.13	18.32	31.62	Positive
102	Negative control	-	-	-	Negative

*: Not Detected

Note: Omsk hemorrhagic fever virus positive material was prepared by mixing synthetic NS2A and C gene materials with the internal control gene hRNase P.

Summary of assay validation results. A total of 100 specimens, including contrived positives (1.5× and 3× LOD), viral RNA references, and bacterial DNA controls, were tested. All OHFV-positive specimens amplified NS2A and C within expected Ct ranges, while negatives showed no OHFV-specific amplification, confirming sensitivity and specificity. The internal control (hRNase P) was consistently detected. The assay achieved 100% PPA (95% CI, 92.9–100.0%) and 100% NPA (95% CI, 92.9–100.0%) with reference results.

Supplementary Tables S8. Repeatability and reproducibility test results obtained by Operator A

<Day 1 test>

No.	Sample ID	1st run (Ct)			2nd run (Ct)			3rd run (Ct)			Final result
		NS2A	C	IC	NS2A	C	IC	NS2A	C	IC	
1	**Precision 1**	32.19	35.35	28.91	32.12	36.07	31.15	32.06	35.54	29.10	Positive
2	**Precision 2**	31.94	35.20	30.72	31.41	32.73	30.27	31.83	32.81	29.87	Positive
3	**Precision 3**	28.42	32.17	29.35	29.04	31.92	31.05	28.94	32.32	29.89	Positive
4	**Precision 4**	-	-	29.15	-	-	29.15	-	-	30.29	Negative

5	**Positive control**	16.97	20.27	16.29							Positive
6	**Negative control**	-	-	-							Negative

No.	Sample ID	NS2A			C			IC		
		Mean Ct.	SD	CV	Mean Ct.	SD	CV	Mean Ct.	SD	CV
1	Precision 1	32.12	0.07	0.21%	35.65	0.37	1.04%	29.72	1.24	4.17%
2	Precision 2	31.73	0.28	0.88%	33.58	1.40	4.18%	30.29	0.43	1.41%
3	Precision 3	28.80	0.34	1.16%	32.14	0.20	0.62%	30.10	0.87	2.88%
4	Precision 4	-	-	-	-	-	-	29.53	0.66	2.23%

<Day 2 test>

No.	Sample ID	1st run (Ct)			2nd run (Ct)			3rd run (Ct)			Final result
		NS2A	C	IC	NS2A	C	IC	NS2A	C	IC	
**1**	Precision 1	32.09	36.00	30.81	32.20	36.04	29.28	32.16	36.34	29.57	Positive
**2**	Precision 2	32.01	35.08	28.60	31.47	34.45	30.78	31.56	34.82	28.81	Positive
**3**	Precision 3	28.98	32.48	31.31	28.88	32.44	30.53	28.86	32.05	30.80	Positive
**4**	Precision 4	-	-	30.74	-	-	30.65	-	-	31.19	Negative
**5**	Positive control	17.06	20.48	16.27							Positive
**6**	Negative control	-	-	-							Negative

No.	Sample ID	NS2A			C			IC		
		Mean Ct.	SD	CV	Mean Ct.	SD	CV	Mean Ct.	SD	CV
**1**	**Precision 1**	32.15	0.05	0.16%	36.12	0.19	0.52%	29.88	0.81	2.71%
**2**	**Precision 2**	31.68	0.29	0.91%	34.78	0.32	0.91%	29.40	1.20	4.08%
**3**	**Precision 3**	28.91	0.07	0.23%	32.32	0.24	0.73%	30.88	0.40	1.28%
**4**	**Precision 4**	-	-	-	-	-	-	30.86	0.29	0.93%

<Day 3 test>

No.	Sample ID	1st run (Ct)			2nd run (Ct)			3rd run (Ct)			Final result
		NS2A	C	IC	NS2A	C	IC	NS2A	C	IC	
**1**	**Precision 1**	32.22	35.58	30.88	32.29	35.64	28.93	32.28	35.45	30.76	Positive
**2**	**Precision 2**	31.87	35.16	30.89	32.00	32.66	30.45	31.45	35.11	30.12	Positive
**3**	**Precision 3**	28.94	31.73	29.61	28.84	32.43	30.49	28.94	31.73	31.00	Positive
**4**	**Precision 4**	-	-	30.23	-	-	29.51	-	-	28.65	Negative
**5**	**Positive control**	17.24	20.41	16.26							Positive
**6**	**Negative control**	-	-	-							Negative

No.	Sample ID	NS2A			C			IC		
		Mean Ct.	SD	CV	Mean Ct.	SD	CV	Mean Ct.	SD	CV
1	Precision 1	32.26	0.04	0.12%	35.56	0.10	0.28%	30.19	1.09	3.62%
2	Precision 2	31.77	0.29	0.91%	34.31	1.43	4.17%	30.49	0.38	1.26%
3	Precision 3	28.91	0.06	0.20%	31.96	0.40	1.27%	30.37	0.70	2.31%

4	Precision 4	-	-	-	-	-	-	29.46	0.79	2.67%

<Day 4 test>

No.	Sample ID	1st run (Ct)			2nd run (Ct)			3rd run (Ct)			Final result
		NS2A	C	IC	NS2A	C	IC	NS2A	C	IC	
1	Precision 1	32.29	35.58	27.68	32.14	36.19	27.72	32.13	36.00	27.93	Positive
2	Precision 2	31.39	32.73	28.39	31.96	34.81	28.29	30.62	34.95	27.86	Positive
3	Precision 3	28.61	32.33	27.77	28.45	32.30	27.73	28.88	31.60	28.25	Positive
4	Precision 4	-	-	28.06	-	-	27.95	-	-	28.36	Negative
5	Positive control	17.11	20.27	16.25							Positive
6	Negative control	-	-	-							Negative

No.	Sample ID	NS2A			C			IC		
		Mean Ct.	SD	CV	Mean Ct.	SD	CV	Mean Ct.	SD	CV
1	Precision 1	32.19	0.09	0.27%	35.92	0.31	0.86%	27.78	0.13	0.48%
2	Precision 2	31.32	0.67	2.15%	34.16	1.24	3.64%	28.18	0.28	1.00%
3	Precision 3	28.65	0.22	0.77%	32.08	0.41	1.29%	27.92	0.29	1.04%
4	Precision 4	-	-	-	-	-	-	28.12	0.21	0.76%

<Day 5 test>

No.	Sample ID	1st run (Ct)			2nd run (Ct)			3rd run (Ct)			Final result
		NS2A	C	IC	NS2A	C	IC	NS2A	C	IC	
1	Precision 1	32.08	35.54	28.44	32.15	35.75	30.63	32.32	36.14	28.53	Positive
2	Precision 2	31.87	32.60	30.23	31.75	35.25	30.57	31.58	34.21	29.87	Positive
3	Precision 3	28.25	31.51	30.33	28.53	32.25	31.21	28.76	31.97	30.06	Positive
4	Precision 4	-	-	30.10	-	-	30.68	-	-	29.72	Negative
5	Positive control	17.11	20.51	16.20							Positive
6	Negative control	-	-	-							Negative

No.	Sample ID	NS2A			C			IC		
		Mean Ct.	SD	CV	Mean Ct.	SD	CV	Mean Ct.	SD	CV
1	Precision 1	32.18	0.12	0.38%	35.81	0.30	0.85%	29.20	1.23	4.23%
2	Precision 2	31.74	0.14	0.46%	34.02	1.34	3.93%	30.22	0.35	1.15%
3	Precision 3	28.51	0.26	0.90%	31.91	0.38	1.18%	30.53	0.60	1.96%
4	Precision 4	-	-	-	-	-	-	30.16	0.48	1.60%

Supplementary Tables S9. Repeatability and reproducibility test results obtained by Operator B

<Day 1 test>

No.	Sample ID	1st run (Ct)			2nd run (Ct)			3rd run (Ct)			Final result
		NS2A	C	IC	NS2A	C	IC	NS2A	C	IC	
1	Precision 1	32.42	35.76	27.19	32.27	36.07	27.52	32.26	36.13	29.52	Positive
2	Precision 2	31.22	34.70	27.33	31.55	35.38	30.22	31.78	35.35	30.89	Positive
3	Precision 3	28.65	31.73	27.28	28.68	32.46	34.12	28.77	31.90	30.68	Positive

4	Precision 4	-	-	26.22	-	-	28.82	-	-	29.15	Negative
5	Positive control	17.19	18.34	24.41							Positive
6	Negative control	-	-	-							Negative

No.	Sample ID	NS2A			C			IC		
		Mean Ct.	SD	CV	Mean Ct.	SD	CV	Mean Ct.	SD	CV
1	Precision 1	32.32	0.09	0.28%	35.98	0.20	0.56%	28.08	1.26	4.49%
2	Precision 2	31.52	0.28	0.90%	35.14	0.38	1.09%	29.48	1.89	6.42%
3	Precision 3	28.70	0.06	0.22%	32.03	0.38	1.19%	30.69	3.42	11.15%
4	Precision 4	-	-	-	-	-	-	28.07	1.60	5.72%

<Day 2 test>

No.	Sample ID	1st run (Ct)			2nd run (Ct)			3rd run (Ct)			Final result
		NS2A	C	IC	NS2A	C	IC	NS2A	C	IC	
1	Precision 1	32.28	36.45	27.04	31.78	36.64	33.22	32.12	36.00	30.01	Positive
2	Precision 2	32.12	35.66	27.15	32.16	32.72	35.95	31.69	32.58	27.99	Positive
3	Precision 3	29.06	32.44	27.47	28.86	32.37	28.28	28.84	32.50	29.97	Positive
4	Precision 4	-	-	27.39	-	-	30.38	-	-	29.82	Negative
5	Positive control	17.20	18.35	27.38							Positive
6	Negative control	-	-	-							Negative

No.	Sample ID	NS2A			C			IC		
		Mean Ct.	SD	CV	Mean Ct.	SD	CV	Mean Ct.	SD	CV
1	Precision 1	32.06	0.25	0.79%	36.36	0.33	0.90%	30.09	3.09	10.27%
2	Precision 2	31.99	0.26	0.81%	33.65	1.74	5.17%	30.36	4.86	16.00%
3	Precision 3	28.92	0.12	0.42%	32.44	0.07	0.21%	28.57	1.28	4.47%
4	Precision 4	-	-	-	-	-	-	29.20	1.59	5.45%

<Day 3 test>

No.	Sample ID	1st run (Ct)			2nd run (Ct)			3rd run (Ct)			Final result
		NS2A	C	IC	NS2A	C	IC	NS2A	C	IC	
1	Precision 1	32.38	36.23	25.69	31.73	36.77	32.50	32.32	36.44	31.19	Positive
2	Precision 2	31.68	35.55	24.06	32.27	32.56	33.34	31.73	35.67	30.06	Positive
3	Precision 3	28.95	32.31	27.33	28.90	32.41	33.27	28.85	32.31	30.24	Positive
4	Precision 4	-	-	27.01	-	-	29.91	-	-	29.78	Negative
5	Positive control	17.34	18.36	27.47							Positive
6	Negative control	-	-	-							Negative

No.	Sample ID	NS2A			C			IC		
		Mean Ct.	SD	CV	Mean Ct.	SD	CV	Mean Ct.	SD	CV
1	Precision 1	32.14	0.36	1.12%	36.48	0.27	0.75%	29.79	3.62	12.14%
2	Precision 2	31.89	0.33	1.03%	34.60	1.77	5.10%	29.16	4.71	16.15%
3	Precision 3	28.90	0.05	0.19%	32.34	0.06	0.17%	30.28	2.97	9.81%

4	Precision 4	-	-	-	-	-	-	28.90	1.64	5.68%

<Day 4 test>

No.	Sample ID	1st run (Ct)			2nd run (Ct)			3rd run (Ct)			Final result
		NS2A	C	IC	NS2A	C	IC	NS2A	C	IC	
1	Precision 1	31.84	36.59	24.55	32.16	35.82	28.54	32.22	36.62	30.28	Positive
2	Precision 2	31.84	34.96	27.44	31.55	34.81	30.66	31.52	34.93	30.32	Positive
3	Precision 3	29.00	32.41	27.22	28.94	32.44	31.84	28.93	32.23	30.58	Positive
4	Precision 4	-	-	27.41	-	-	29.51	-	-	30.12	Negative
5	Positive control	31.84	36.59	24.55	32.16						Positive
6	Negative control	-	-	-							Negative

No.	Sample ID	NS2A			C			IC		
		Mean Ct.	SD	CV	Mean Ct.	SD	CV	Mean Ct.	SD	CV
1	Precision 1	32.07	0.20	0.64%	36.35	0.45	1.25%	27.79	2.93	10.56%
2	Precision 2	31.63	0.18	0.56%	34.90	0.08	0.22%	29.47	1.77	6.00%
3	Precision 3	28.96	0.04	0.14%	32.36	0.11	0.35%	29.88	2.39	8.00%
4	Precision 4	-	-	-	-	-	-	29.01	1.42	4.91%

<Day 5 test>

No.	Sample ID	1st run (Ct)			2nd run (Ct)			3rd run (Ct)			Final result
		NS2A	C	IC	NS2A	C	IC	NS2A	C	IC	
1	Precision 1	31.69	36.43	23.78	32.30	35.91	27.65	32.34	36.07	29.78	Positive
2	Precision 2	32.26	34.78	27.39	32.22	32.82	29.08	31.43	32.51	27.53	Positive
3	Precision 3	29.06	32.33	26.78	29.17	32.08	31.13	29.37	32.13	30.45	Positive
4	Precision 4	-	-	27.44	-	-	28.81	-	-	30.34	Negative
5	Positive control	17.66	18.38	25.35							Positive
6	Negative control	-	-	-							Negative

No.	Sample ID	NS2A			C			IC		
		Mean Ct.	SD	CV	Mean Ct.	SD	CV	Mean Ct.	SD	CV
1	Precision 1	32.11	0.36	1.13%	36.14	0.27	0.74%	27.07	3.04	11.23%
2	Precision 2	31.97	0.47	1.46%	33.37	1.23	3.68%	28.00	0.94	3.35%
3	Precision 3	29.20	0.16	0.54%	32.18	0.13	0.41%	29.45	2.34	7.93%
4	Precision 4	-	-	-	-	-	-	28.87	1.45	5.03%

Supplementary Tables S10. Interference study results.

Specimen type	Threshold cycle (Ct)								
	1st run (Ct)			2nd run (Ct)			3rd run (Ct)		
	NS2A	*C*	**IC**	*NS2A*	*C*	**IC**	*NS2A*	*C*	**IC**
Phosphate-buffered saline (PBS)-spiked specimen	31.89	36.84	26.59	32.84	36.50	27.19	32.10	35.63	27.42

2 mM ethylene diamine tetraacetic acid (EDTA)-spiked specimen	32.65	36.81	28.33	32.11	35.68	28.79	32.12	35.45	29.23
Viral transport medium (VTM)-spiked specimen	32.57	35.69	27.13	32.55	35.66	27.54	32.34	35.60	27.73
Neat specimen (no additive)	32.33	36.50	26.53	32.24	35.92	26.68	32.88	35.29	26.74
Negative matrix specimen (normal human serum)	-*	-	28.43	-	-	26.67	-	-	26.74
Positive control	17.29	19.42	34.50						
Negative control	-	-	-						

Specimen type	*NS2A*			*C*			IC		
	Mean Ct	SD	%CV	Mean Ct	SD	%CV	Mean Ct	SD	%CV
PBS-spiked specimen	32.28	0.50	1.55%	36.33	0.62	1.71%	27.07	0.43	1.58%
2 mM EDTA-spiked specimen	32.29	0.31	0.96%	35.98	0.73	2.03%	28.78	0.45	1.56%
VTM-spiked specimen	32.49	0.13	0.39%	35.65	0.05	0.13%	27.47	0.31	1.12%
Neat specimen (no additive)	32.48	0.35	1.06%	35.90	0.61	1.70%	26.65	0.11	0.42%
Negative matrix specimen (normal human serum)	-	-	-	-	-	-	27.28	1.00	3.66%

* : Not Detected
